# The role of carbonic anhydrase IX in cancer development: links to hypoxia, acidosis, and beyond

**DOI:** 10.1007/s10555-019-09799-0

**Published:** 2019-05-10

**Authors:** Silvia Pastorekova, Robert J Gillies

**Affiliations:** 1 Department of Tumor Biology, Institute of Virology, Biomedical Research Center, University Science Park for Biomedicine, Slovak Academy of Sciences, Dúbravská cesta 9, 845 05 Bratislava, Slovakia; 2 Department of Cancer Physiology, H. Lee Moffitt Cancer Center, 12902 Magnolia Avenue, Tampa, FL 33612 USA

**Keywords:** Carbonic anhydrase IX, pH regulation, Tumor microenvironment, Hypoxia, Acidosis, Cancer progression

## Abstract

Cancer development is a complex process that follows an intricate scenario with a dynamic interplay of selective and adaptive steps and an extensive cast of molecules and signaling pathways. Solid tumor initially grows as an avascular bulk of cells carrying oncogenic mutations until diffusion distances from the nearest functional blood vessels limit delivery of nutrients and oxygen on the one hand and removal of metabolic waste on the other one. These restrictions result in regional hypoxia and acidosis that select for adaptable tumor cells able to promote aberrant angiogenesis, remodel metabolism, acquire invasiveness and metastatic propensity, and gain therapeutic resistance. Tumor cells are thereby endowed with capability to survive and proliferate in hostile microenvironment, communicate with stroma, enter circulation, colonize secondary sites, and generate metastases. While the role of oncogenic mutations initializing and driving these processes is well established, a key contribution of non-genomic, landscaping molecular players is still less appreciated despite they can equally serve as viable targets of anticancer therapies. Carbonic anhydrase IX (CA IX) is one of these players: it is induced by hypoxia, functionally linked to acidosis, implicated in invasiveness, and correlated with therapeutic resistance. Here, we summarize the available experimental evidence supported by accumulating preclinical and clinical data that CA IX can contribute virtually to each step of cancer progression path *via* its enzyme activity and/or non-catalytic mechanisms. We also propose that targeting tumor cells that express CA IX may provide therapeutic benefits in various settings and combinations with both conventional and newly developed treatments.

## Introduction

Solid tumors often contain regions of hypoxia and/or acidosis. These microenvironmental stresses create selective pressure in favor of adaptable tumor cells that undergo massive molecular and phenotypic changes associated with cancer progression and treatment resistance [1–3]. This important connection predisposes the molecules involved in adaptation to hypoxia and acidosis to serve as prognostic indicators, predictive factors, and/or targets for anticancer therapy that deserve increasing attention.

## Hypoxia

Hypoxia is a key factor of the tumor microenvironment caused by regional diffusion and perfusion thresholds of oxygen caused primarily by aberrant tumor vasculature. Hypoxia can fluctuate from moderate to severe, acute to chronic, and intermittent to persistent, and induce a spectrum of cellular responses leading to aggressive tumor phenotypes [1, 3]. At the molecular level, these changes are principally determined by hypoxia-inducible factor (HIF)–mediated reshaping of the transcriptional profile, which depends on the extent, duration, and severity of hypoxia and by unfolded protein response (UPR)–modified translational program activated in conditions of severe hypoxia/anoxia [4, 5]. HIFs (1 and 2) operate *via* their oxygen-dependent α subunits, which are modified by oxygen-requiring prolyl hydroxylases and directed to degradation in proteasome by pVHL tumor suppressor protein under normoxic conditions, while they escape degradation and are stabilized and activated in hypoxia. Following dimerization with a constitutive β subunit, HIF transcription factors bind to hypoxia-response elements in regulatory regions of a multitude of genes and activate or induce their transcription [4]. However, normoxic elevation and activation of HIF can be caused either by loss/inactivating mutations in VHL (occurring in most clear cell renal cell carcinomas) or by oncogenic pathways that increase the transcription, translation, and/or activity of the HIF-α subunit in non-RCC tumors [6].

HIF targets include genes encoding mediators of angiogenesis such as vascular endothelial growth factor (VEGF) and VEGF receptors, enzymes of the glycolytic pathway such as hexokinase 2, lactate dehydrogenase, and glucose transporters (GLUT-1, GLUT-3), as well as CA IX. In all, the HIF-mediated response to hypoxia is a coordinated and temporally regulated response involving genes that regulate erythropoiesis, vascular remodeling and plasticity, cell proliferation and viability, cell adhesion, cell matrix metabolism, pH regulation, *etc.* Importantly, hypoxia has serious clinical consequences and its occurrence in tumor tissues has been clearly associated with cancer progression, metastasis, and resistance to chemo-, radio-, and immuno-therapies [1, 6].

## Acidosis

The hypoxia-triggered metabolic shift toward glycolysis allows for the sustained, albeit less efficient production of energy in conditions of reduced or absent oxygen, a substrate of oxidative phosphorylation [7]. This is critical to survival of hypoxic tumor cells. Hypoxia also selects for inherently glycolytic cells developed through oncogenic events. Importantly, glycolysis not only generates energy but can also contribute to the synthesis of biomass (e.g. nucleotides, amino acids, and lipids) required for the production of new cells during tumor expansion. Notably, tumor cells rely on fermentative glycolysis even in the presence of oxygen, a phenomenon known as aerobic glycolysis, or the Warburg Effect [8]. Tumor cells also depend on glutaminolysis, which can feed the mitochondrial TCA cycle and pentose phosphate pathway and thereby contribute to synthesis of fatty acids, nonessential amino acids, and nucleosides [9].

Oncogenic metabolism of tumor cells shared to a variable extent among respiration, glycolysis, and glutaminolysis generates an excess of acidic metabolic end products, including lactic acid, protons, and carbon dioxide. To avoid cytosolic accumulation of these acidic metabolites and prolonged intracellular acidosis, cells redirect the transmembrane ion fluxes and enhance activity of pH-regulating machinery [10]. Many constituents of this machinery and their upstream regulators are pH-sensitive molecules and are thus activated once the intracellular pH (pHi) reaches acidic values incompatible with the biosynthetic reactions and signaling. Their purpose appears to be to return pHi to slightly alkaline values that are more favorable to cell survival and proliferation. Elimination of intracellular acidosis generally occurs through diffusion of CO_2_, export of lactate and protons, and through the import of bicarbonate ions produced by the hydration of CO_2_ [11]. However, this leads to pericellular acidosis that often persists in tumor microenvironment because the acidic metabolic waste cannot be effectively removed by the abnormal tumor vasculature [12].

Tumor cells with activated pH-regulating machinery can resist toxic effects of extracellular acidosis generated by oncogenic metabolism and even benefit from acidosis-supported acquisition of more aggressive tumor phenotypes. Therefore, they possess selective advantage against surrounding normal cells that cannot adapt [13].

Similar to hypoxia, acidosis is associated with resistance to chemo-, radio- and immune-therapies. Indeed, acidosis is a potent inhibitor of T cell effector functions [14] and neutralization of tumor acidity can improve response to immunotherapy [15, 16]. Acidosis also influences tumor metabolic preferences, reducing glycolysis while promoting mitochondrial activity. Acidosis supports progression-related phenomena such as angiogenesis, invasion, and metastasis and is linked with cellular phenomena including aneuploidy and mutation rate, autophagy and survival, cell migration, and release of exosomes [17, 18]. Rohani et al. [19] have recently demonstrated that acidosis is enriched at tumor-stroma interfaces (in addition to regions of hypoxia) and that cells within the acidic front are invasive and proliferative. Consistent with previous observations [20], these acidic regions were associated with upregulated expression of CA IX.

## Carbonic anhydrase IX

Carbonic anhydrase IX (CA IX) is a tumor-associated, cell-surface glycoprotein that is induced by hypoxia, involved in adaptation to acidosis and implicated in cancer progression *via* its catalytic activity and/or non-catalytic functions.

**Fig. 1:**
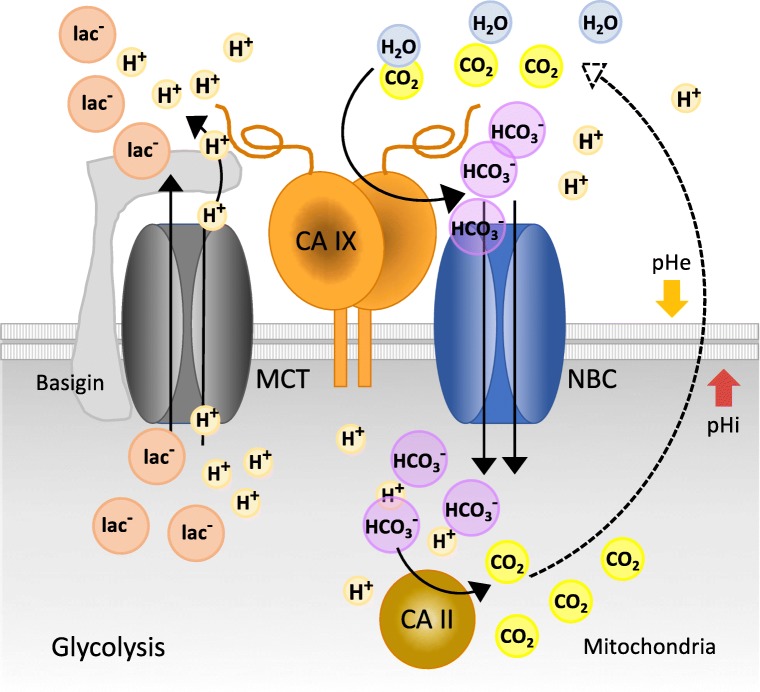
Schematic model of the CA IX role in pH regulation in hypoxic cancer cells. CA IX can cooperate with bicarbonate transporters (NBC) as well as monocarboxylate transporters (MCT) to remove acid from the intracellular space in order to secure cell survival. In bicarbonate transport metabolon (on the right side), CA IX acts *via* its extracellular enzyme domain that catalyzes a conversion of pericellular carbon dioxide to protons and bicarbonate ions. Bicarbonate ions are uploaded by the adjacent bicarbonate transporters and transported across the plasma membrane to the cytoplasm. Inside the cell, bicarbonate reacts with intracellular protons resulting from diverse metabolic paths. This reaction (possibly catalyzed by the cytoplasmic CA II isoform) results in their conversion to CO_2_, which leaves the cell by diffusion. Consumption of the intracellular protons by the imported bicarbonate ions helps to increase the intracellular pH to the values permissive for metabolic processes, signaling, and proliferation. On the other hand, extracellular protons generated by the same CA IX–catalyzed reaction remain outside of the cell and contribute to acidification of the pericellular milieu. CA IX can also contribute to lactate export (on the left side) by a non-catalytic mechanism that includes its cooperation with MCT-basigin complex and an employment of its highly acidic N-terminal PG domain as an antenna driving the MCT-mediated proton flux in parallel with lactate extrusion out of the cell. This causes further extracellular acidification, which supports invasion of cancer cells to the surrounding normal tissue

CA IX belongs to the α carbonic anhydrase family of zinc metalloenzymes that catalyze the reversible hydration of carbon dioxide to bicarbonate ions and protons [21]. This simple reaction is essential for virtually all biological processes requiring acid-base balance in subcellular compartments and across the plasma membrane. There are 15 human CA isoforms; out of which, 3 are inactive and 12 range in activity from weak to very strong. Most of the isoenzymes are predominantly expressed in differentiated cells to fulfill specialized roles in various tissues and organs, such as production of gases, body fluids, bone resorption, and biosynthetic reactions [22]. CA IX is one of three exofacial CA isoforms, along with CA IV and CA XII, and has a unique position in this enzyme family due to its strong association with cancer, hypoxia-related expression pattern, acidic pKa optimum, and inclusion of an extra proteoglycan-like domain protruding from the globular catalytic domain of the enzyme, which is anchored in the plasma membrane *via* a single-pass transmembrane region and a short cytoplasmic tail [23–25]. The CA IX enzyme active site in the catalytic domain is facing the extracellular space and by accelerated CO_2_ hydration contributes to pH regulation across the plasma membrane, simultaneously facilitating CO_2_ diffusion and proton mobility in the tumor tissue [11, 26, 27]. It is now well established that it does so in a spatial and functional cooperation with diverse acid extruders and bicarbonate importers (Fig. 1), including sodium-dependent bicarbonate transporters NBCe1 and NBCn1 [28–30], lactate and proton-exporting monocarboxylate transporters MCT1 and MCT4 [31], sodium/hydrogen exchanger (NHE1), and other ion exchanges, pumps, and transporters (unpublished data). Although CA IX catalytic activity is key to these processes, PG-like domain can also act *via* a non-catalytic mechanism, in which it serves as an antenna enhancing export of protons coupled with facilitated export of lactate ions through monocarboxylate transporters [32]. Involvement of CA IX in pH regulation has multiple consequences supporting tumor phenotype as discussed below.

Besides being a pH regulator, CA IX can also behave as an adhesion molecule. Its PG-like domain contributes to the assembly and maturation of focal adhesion contacts during cell attachment and spreading on solid supports [33]. Conversely, CA IX can destabilize intercellular adhesion contacts by disconnection of E-cadherin from the cytoskeletal anchorage through the competitive binding to beta catenin [34]. It is not yet clear, whether in these processes CA IX acts solely *via* mechanosensitive adhesion forces or whether it also involves pH control mechanisms. However, it has been recently demonstrated that low tumor pH can downregulate E cadherin expression and/or induce its cleavage, and depending on the timing of exposure, acidosis can decrease or increase cancer cell adhesion [35].

## CA IX regulation and expression pattern

CA IX is one of the best responders to low oxygen (ranging from anoxia to moderate hypoxia), mainly because the CA IX-encoding gene (according to the nomenclature designated as *CA9*) is transcriptionally regulated through HIF-1 binding to HRE consensus sequence localized just in front of the transcription initiation site, under conditions of HIF-mediated nucleosomal disassembly [36, 37]. For full transcriptional activation of *CA9*, HIF cooperates with the SP1 transcription factor that appears to mediate the *CA9* induction by increased cell density and by acidosis (both normoxic and hypoxic) in a cell type–specific manner [38, 39]. Since expression and activation of HIF are affected by oncogenic signaling, transcription of the *CA9* gene also increases in response to activation of the MAPK and PI3K pathways and upstream tyrosine kinases, including SRC and RET [40–42]. Expectedly, inactivation of the pVHL tumor suppressor protein, which negatively controls HIF stability, results in the constitutive elevation of *CA9* gene expression in renal cell cancers, RCC [36, 43, 44]. Moreover, hypoxia regulates splicing of the CA IX mRNA and a PKA-mediated phosphorylation of the cytoplasmic tail of the CA IX protein, in both cases affecting its enzyme activity [45, 46]. Additional post-translational modifications of the extracellular domain of CA IX include N-glycosylation by high mannose sugar chain in the catalytic domain and O-glycosylation by heparan or chondroitin sulfate glycosaminoglycan chains in the N-terminal PG-like region [21, 47]. Impact of these modifications on the functioning of CA IX in pH regulation and cell adhesion has not been fully clarified, although glycosaminoglycan modification was shown to attenuate antibody-induced internalization of CA IX *via* increased association with caveolin-1 clusters in acidosis-sensitive membrane raft domains [47].

In this connection, it should be mentioned that CA IX can internalize from the cell surface to the cell interior *via* endocytosis. This can be induced by physiological stresses including hypoxia and calcium depletion as well as by specific antibodies binding to its extracellular domain [34, 48, 49]. Further, the ectodomain of CA IX can be cleaved by metalloproteinase ADAM17 and released to the microenvironment in response to hypoxia and acidosis, as well as toxic insults of carbonic anhydrase inhibitors or chemotherapeutic drugs [50, 51]. CA IX is also released to the extracellular milieu as a component of exosomes [52, 53]. All of these processes can lead to depletion of CA IX from the surface of tumor cells but, on the other hand, generate potential messengers of autocrine and/or paracrine signaling. This aspect of CA IX regulation clearly needs further investigations.

The dominant role of hypoxia in the control of CA IX expression is reflected by its presence in a broad range of solid tumors and by its distribution in tumor tissues that can be either diffuse in RCC due to VHL defect-mediated constitutive hypoxia-like response (pseudohypoxia) or regional in other tumor types due to HIF pathway activation by physiological hypoxia. Since CA IX responds to hypoxia ranging from moderate to strong and persists hours to days after reoxygenation due to high protein stability [54], its regional distribution only partially overlaps with that of other hypoxia-regulated molecules including HIF-1α, VEGF, and GLUT1*.* CA IX expression pattern is also shaped by acidosis, which extends beyond the hypoxic regions [19].

CA IX expression in non-cancerous tissues is rare and generally confined to epithelia of the stomach, gallbladder, pancreas, and intestine [55]. CA IX–deficient mice display hyperplasia of the stomach mucosa associated with loss of parietal cells, impaired basolateral pH regulation, perturbed barrier functions, and chronic inflammation [56, 57]. This supports the view that CA IX is a part of a defense mechanism protecting gastric epithelia from acid load. So far, it remains unclear, which factors drive CA IX expression in non-transformed cells, but the gastric physiology suggests a role for acidosis, inflammation, and even hypoxia that occurs in all mucosal cells of aging stomach due to decreasing mucosal blood flow [58]. Thus, hypoxia and acidosis seem to be universal drivers of CA IX expression independently of cell phenotype.

## CA IX contributions to key steps of cancer development

**Fig. 2:**
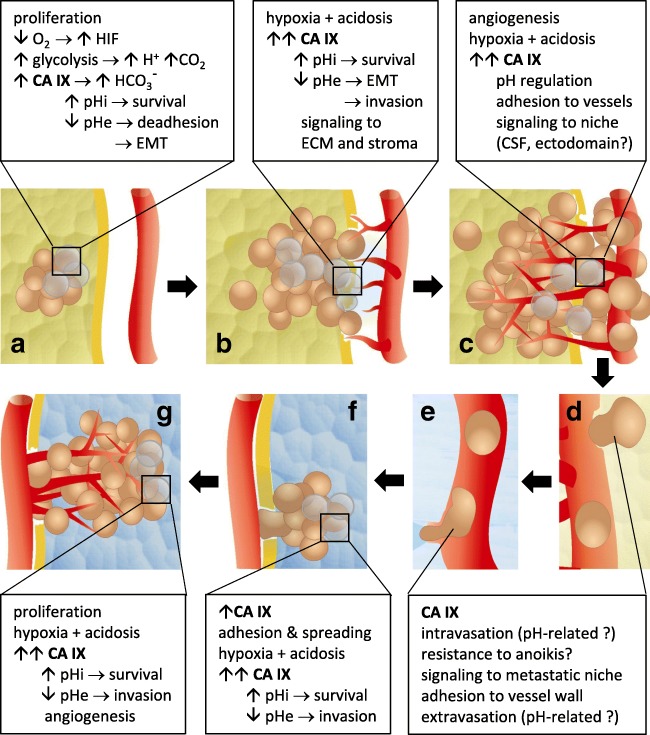
CA IX involvement in various steps of cancer progression. (A) In ducal carcinoma *in situ*, CA IX expression is induced by local hypoxia and *via* regulation of pH participates in adaptation to metabolism generating excess of acidic products. This allows for cancer cell survival and proliferation. (B, C) In the growing tumor, CA IX further protects cancer cells from hypoxia and intracellular acidification. Moreover, *via* exacerbating extracellular acidosis, CA IX appears to contribute to angiogenesis, ECM degradation, epithelial-mesenchymal transition and invasiveness, tumor-stroma crosstalk, and tumor-to-niche signaling. (D, E) CA IX can potentially mediate adhesion of cancer cells to vessels and *via* generating local acidosis allows for transmigration to the lumen. In circulation, CA IX can presumably protect the cells from anoikis and then facilitate their extravasation to the site of secondary residence. (F) Homing of metastatic lesion can be facilitated by CA IX–assisted formation of focal adhesion contacts and cell spreading, and initial growth of metastasis takes advantage from CA IX-mediated pH regulation. (G) Expansion of metastasis recapitulates the situation in primary tumor with possible role of CA IX in protection of cells from hypoxia and acidosis. All of these “points of action” of CA IX offer opportunities for its therapeutic targeting

Accumulating experimental evidence suggests that CA IX is functionally involved in diverse aspects of cancer development (Fig. 2).

### DCIS

In earliest stages of carcinogenesis, hyperplastic epithelia are confined to growing inside of ducts by basement membranes. As these cells proliferate, they grow into the ductal lumens and further from the stroma and the blood supply. As the diffusion distance of oxygen in tissues is 160–200 μm, peri-luminal cells quickly become hypoxic, and acidotic, which form strong evolutionary selection forces for cells that can survive these harsh conditions [59]. Notably, CA IX is highly expressed in peri-luminal areas in DCIS and is highly associated with necrosis and grade [60]. Given the role of CA IX in regulating intracellular pH, it can be surmised that CA IX plays an important role in survival of cells within DCIS. Further, by promoting extracellular acidification [27], it also contributes heavily to the evolutionary dynamics of DCIS progression to locally invasive disease.

### Primary tumor growth

Growing tumor tissue is characterized by increased proliferation, adaptation to microenvironmental stresses (hypoxia, acidosis, deprivation of nutrients), angiogenesis, and invasion. CA IX directly participates in these hallmarks, as supported by the fact that its suppression, mutation/deletion, pharmacologic inhibition, or treatment with monoclonal antibodies result in significantly reduced growth of tumor xenografts *in vivo* [48, 61–64]. Experimental evidence from diverse cell models indicates that CA IX acts here primarily *via* its catalytic activity and pH regulating function, which helps to maintain slightly alkaline intracellular pH that is critical for survival, metabolic performance, and proliferation of cancer cells, particularly in hypoxic conditions. Simultaneously, CA IX exacerbates extracellular acidosis that can activate proteases to cleave extracellular matrix, facilitate epithelial-mesenchymal transition and invasion, reprogram metabolism, affect cell adhesion, and support inflammation and angiogenesis. CA IX has also been proposed to support angiogenesis as a component of exosomes promoting migration of endothelial cells and tube formation [52]. Suppression of CA IX leads to reduced expression of ECM components including collagen IV, and the MMP2 and MMP9 proteases [65]. CA IX deficiency is also associated with reduced migration and invasive propensity, while overexpression has opposite effects [30]. It was shown that CA IX re-localizes to protruding fronts of migrating cells together with other constituents of pH regulating machinery and facilitates formation of reverse pH gradient needed for remodeling of cytoskeleton and cell body movement [30, 66]. CA IX is present in invadopodia, where it interacts with MMP14, integrins, bicarbonate transporter NBCn1, and other transport proteins needed for effective invasion [67]. CA IX also participates in communication between tumor and stroma. Its upregulation in cancer-associated fibroblasts (CAFs) in response to ROS-mediated stabilization of HIF-1 in normoxia leads to extracellular acidosis and activation of epithelial-mesenchymal transition (EMT) in epithelial cancer cells [68]. In line with these observations, CA IX expression in invasive tumor lesions was detected at the tumor-host interface in cells that exhibit invasion-promoting, rapidly proliferating phenotypic properties [19, 20]. In this respect, it would be interesting to investigate whether CA IX can facilitate collective migration/invasion of cancer cells that shows higher pro-metastatic potential [69].

Earlier studies suggest that the CA IX ectodomain can mediate paracrine signaling *via* binding to the surface of dendritic cells (DC) and potentially modulate their immune responses [70]. This might be particularly interesting in light of the fact that hypoxia (and also acidosis) promotes the recruitment and pro-inflammatory phenotype in DCs within tumor tissue [71].

### Metastatic dissemination of cancer cells

The metastatic process involves discrete steps of intravasation, survival in circulation, and extravasation. During intravasation and extravasation, cancer cells have to attach to the vessel wall and transmigrate to and from the vessel lumen. This attachment might be mediated by PG-like domain of CA IX, since it is modified by sulfated sugar chains that can potentially interact with ECM and vascular cells [47]. However, it cannot be excluded that extracellular acidosis locally created by CA IX catalytic activity causes disconnection of cell-cell junctions between vascular cells and degradation of the extracellular matrix, thereby supporting transit of tumor cells across the vessel wall [72]. In circulation, CA IX appears to protect tumor cells from anoikis, but the mechanism is not known. It can be speculated that CA IX–contributed acidosis promotes a stem phenotype leading to release of cancer cell clusters from the primary tumor mass. The cell clusters then enter circulation and allow for survival in non-adherent conditions and metastasis [73, 74].

### Homing and growth of metastatic lesions

Following extravasation, tumor cells need to attach and spread in secondary site, proliferate to form metastatic lesions, and survive microenvironmental stresses generated in growing metastatic tissue. Experimental data from *in vivo* models suggest that metastatic lesions derived from xenografted tumor cells do express CA IX and this is supported by available (although rare) studies of human tumor tissues [75–78].

Both PG-like and CA domains appear to be involved in successful colonization. CA IX can promote metastasis by pH-dependent NF-κΒ activation leading to secretion G-CSF and mobilization of granulocytic myeloid–derived suppressor cells to aid in establishment of a pre-metastatic niche [79]. CA IX-mediated cell attachment and spreading depends on the presence and integrity of the PG-like domain [33]. Deletion or blocking of PG with a monoclonal antibody reduces cell adhesion to solid support, lowers lactate/proton extrusion, and decreases cell proliferation [32, 33]. On the other hand, survival of microenvironmental stresses including hypoxia and acidosis depend on the enzyme activity of the catalytic domain as described above. It is believed that metastatic lesions are generated by cancer stem cells and CA IX expression was shown to be associated with stem-like phenotype [80, 81].

## Translation into the clinic

Current literature offers more than 1000 papers that explore clinical value of CA IX. The vast majority of data suggest that CA IX can serve as a biomarker and/or therapy target that can be potentially employed in diverse tumor types and settings, for more details see [82]. In this respect, it is important to discriminate between tumors that express CA IX as a consequence of the inactivating mutation of pVHL tumor suppressor protein and tumors in which CA IX is present in connection with microenvironmental hypoxia and/or acidosis. The former category is represented primarily by the clear cell renal cell carcinoma (ccRCC) that often carry an inactivating mutation/deletion of the VHL tumor suppressor gene and display “constitutive” HIF stabilization and activation of HIF-regulated genes including CA IX [83]. This leads to expression of CA IX in more than 90% of these carcinomas, in which CA IX can be detected in a high percentage of tumor cells [44]. However, expression of CA IX decreases in more advanced ccRCC stages due to switch from the HIF-1 to the HIF-2 isoform. Accordingly, it has been shown that CA IX IHC staining fewer than 85% of cells is a poor prognostic marker [84]. However, the fraction of CA IX positive cells is still relatively large to provide a targetable terrain.

The situation is distinct in many other tumor types, where CA IX is expressed regionally in areas that are hypoxic and/or acidic (as explained above) and usually increases with increasing tumor stage and grade. CA IX staining is often present in broad perinecrotic zones including moderately hypoxic and viable tumor cells with metastatic potential [36]. Notably, this only partially overlaps with the distribution of chemical marker of hypoxia pimonidazole, HIF and other endogenous biomarkers of hypoxia (VEGF, GLUT-1, MCT-4) due to differences in hypoxic thresholds for induction, post-translational stabilities and additional regulatory factors at all expression levels [85]. Recent studies also show that CA IX is present in areas that are acidic and overlap with markers of acidosis, including fluorescent peptides and CA inhibitors selectively binding to CA IX [19, 76].

A majority of the published studies (excluding ccRCC) report on head and neck carcinoma, breast carcinoma, brain tumors, lung, and colorectal carcinoma. CA IX is mostly stained at the plasma membrane of tumor cells. Cytoplasmic and/or nuclear staining signals can be occasionally seen, but their biological meaning is not clear. CA IX expression in tumor tissues associates with various prognostic factors, including c-ErbB2/HER2, EGFR, MUC-1, MMP9. ostepontin, CD44, LOX, Ki-67, cyclin E, bcl-2, and c-MET, for more details refer to [82]. CA IX can be detected also in tumor stroma, where it has been associated with poor prognosis [86]. Finally, in agreement with the experimental evidence on the ectodomain shedding and release in exosomes, CA IX can be detected in body fluids of cancer patients, reviewed in [82]. This fact can be clinically exploited for noninvasive screening or monitoring, but the data available so far are inconclusive, in part due to use of incompatible detection platforms [87].

Clinical correlates of CA IX expression are tumor-type- and context-dependent. A meta-analysis of selected papers published between 2001 and 2015, and encompassing more than 24 thousand patients with non-RCC tumors, was performed by van Kuijk et al. [88]. It revealed strongly significant associations between CA IX expression evaluated by immunohistochemistry and all endpoints: overall survival, disease-free, locoregional control, disease-specific, metastasis-free survival, and progression-free survival. Subgroup analyses showed similar associations in the majority of tumor sites and types. In conclusion, these results show that patients having tumors with high CA IX expression have higher risk of disease progression, and development of metastases, independent of tumor type or site. In addition, there are numerous studies showing correlation between CA IX positivity and resistance to chemotherapy, radiotherapy, and even immunotherapies directed to other cancer-related molecular targets, such as HER-2, VEGF, and PD-1 (see below). These findings support the usefulness of clinical tests determining patient’s prognosis and therapy outcome based on CA IX expression and provide a rationale for the development of new CA IX–targeted treatment strategies.

Detection of CA IX for prognostic and predictive purposes in routine clinical settings can be performed preferably by immunohistochemistry using specific monoclonal antibodies. According to meta-analysis described above [88], the most often used antibody is M75, which enabled identification of the CA IX protein (initially named MN) and cloning of the *CA9* cDNA and gene [21, 23, 89]. M75 recognizes a linear epitope in the N-terminal PG-like domain of CA IX that is not affected by denaturation even during a long-term storage of archived tissue specimens [90]. On the other hand, monoclonal antibodies directed to the catalytic domain of CA IX, including G250, VII/20, and MSC8, recognize conformational epitopes that are disrupted in reducing and denaturing conditions [91–93]. Although these CA-domain-specific antibodies are not suitable for routine immunohistochemistry on paraffin-embedded tissue sections, they can be employed for *in vivo* imaging (such as cG250-derived REDECTANE or GIRENTUXIMAB), blocking CA IX catalytic activity (such as MSC8) and CA IX–targeted immunotherapy. Noteworthy, radiolabeled cG250 can visualize primary and metastatic tumor lesions in ccRCC patients [94–96]. Literature describes additional CA IX–specific antibodies produced by diverse screening approaches [97] and companies offer numerous CA IX antibody products, sometimes generating inconsistent data [98]. Careful selection of the antibodies for detection and/or targeting is therefore of key importance for better understanding and clinical exploitation of CA IX. Promising clinical uses of CA IX include molecular imaging *in vivo*, which currently attracts a lot of attention and includes approaches using diverse imaging agents based on monoclonal antibodies, inhibitors, and other compounds labeled by various radionuclides [99–102].

With regard to therapeutic applications, two basic CA IX-targeting strategies have been under development since the early era of the CA IX research and involve many R&D efforts covered by a number of international patents.

The first CA IX–targeting strategy builds on the role of CA IX in pH regulation and exploits compounds that inhibit the CA IX enzyme activity through binding at or near its active site, thereby compromising the CA IX catalytic function. This strategy is currently in preclinical and/or early clinical stages (https://clinicaltrials.gov/ct2/results?term=cancer+AND+carbonic+anhydrase+IX&Search=Search). The second approach for targeting CA IX takes advantage of tumor-related distribution of CA IX and is based on utilization of specific monoclonal antibodies to detect and cause selective killing of tumor cells that express CA IX. The mechanism of such immunotherapy can include either activation of cytotoxic immune response (particularly antibody-dependent cellular cytotoxicity, ADCC) or delivery of toxic drugs as antibody-drug conjugates, ADC. One line of this strategy has already undergone phase III clinical testing with promising but still not definitive results as discussed below, while additional lines are in preclinical and early clinical development (see the link above).

Inhibitors of the carbonic anhydrase enzyme activity represent emerging anticancer drugs as thoroughly reviewed elsewhere [103, 104]. Different groups of sulfonamides, sulfamates, and related compounds with modifications conferring selectivity and/or membrane impermeability can efficiently inhibit CA IX *in vitro* and some of these show anticancer effects in xenografted subcutaneous or metastatic animal models [63, 105]. Interestingly, certain clinically used inhibitors of tyrosine kinases and metabolic enzymes as well as natural compounds can inhibit CA IX [106]. However, clinical use of CA IX inhibitors is complicated by the risk of unwanted adverse effects and compensation mechanisms evolving in cancer cells as a result of their phenotypic plasticity. Thus, targeting CA IX function alone may not be sufficient to achieve a satisfactory therapeutic effect, and therefore, approaches leading to dual effects or synthetic lethality are being explored. One such example used an anti-VEGF therapy followed by the inhibition of CA IX activity [107], which was based on the observation that anti-angiogenic therapy exacerbates intratumoral hypoxia leading to induction of CA IX as a survival mechanism. In addition, dual targeting of the bioreductive nitroimidazole-based anti-CA IX sulfamide drug DH348 was shown to reduce tumor growth in mice and sensitize tumors to irradiation in a CA IX–dependent manner [108].

The second main CA IX–targeting strategy based on immunotherapy exploits the tumor-related expression pattern of CA IX. This approach using monoclonal antibodies ensures high specificity and selectivity toward CA IX that is currently not achievable with chemical compounds. In case of ADCC as the main mechanism of action, the killing effects is fast and thus forestalls development of compensatory mechanisms. Most of the studies, including the clinical trials using this strategy, were performed in RCC animal models and in patients with non-metastatic RCC using the chimeric human-mouse monoclonal antibody G250 known under commercial names RENCAREX® or GIRENTUXIMAB® [94]. The antibody was found safe and well tolerated, and in a subgroup of patients with high tumor CA IX scores showed a prolonged disease-free survival up to 22 months [109]. There have been also attempts to develop CA IX antibody-drug conjugates, but in these approaches, both linker and drug are equally important as the antibody itself and all three components together determine the outcome of therapy and potential side effects, as it was in the case of the human 3ee9 antibody conjugated to monomethyl auristatin E through a self-cleavable linker (BAY 79-4620), which showed potent antitumor efficacy in several xenograft models [110], but failed in a clinical trial due to inacceptable toxicity.

CA IX expression in non-RCC tumors is less frequent and more heterogeneous, with much lower fraction of the CA IX–positive cells in tumor tissue. However, its associations with a pro-metastatic phenotype and therapy resistance make it an attractive target especially in tumor types characterized by highly aggressive behavior, short survival, and absence of effective treatment options. Immunotherapeutic strategies may include targeting tumor cells that survived primary therapy protocols as suggested by the observations that non-responders to standard chemotherapy and radiotherapy show increased CA IX expression [111]. Since tumor cells residing in regions of hypoxia and/or acidosis (and hence expressing CA IX) are inherently associated with therapy resistance, it is conceivable that chemotherapy/radiotherapy will leave these cells alive and permit their proliferation in metastatic lesions. Of course, this assumption needs more experimental and clinical evidence, as there are only few data on detection of CA IX directly in metastatic lesions of non-RCC patients [75–78].

An independent approach with potential immunomodulating effect employs autologous dendritic cells (DCs) transduced with a replication-defective adenoviral vector carrying the fusion gene (GMCA-9) encoding granulocyte-macrophage colony-stimulating factor (GM-CSF) and carbonic anhydrase IX (CA-IX or CA9). The autologous DCs are transduced *ex vivo* and express the GMCA-9 fusion protein on the cell surface. Upon intradermal administration of the AdGMCAIX-transduced autologous DCs back into the patient, the DCs are expected to activate a cytotoxic T lymphocyte–mediated response against tumor cells positive for the CA9 antigen, and generate memory T cells, potentially resulting in decreased tumor growth. This strategy is currently in the phase 1 clinical trial to determine the safety and tolerability in patients with metastatic renal cell carcinoma [112].

## Conclusions

Currently available data on CA IX support its intimate connection with tumor hypoxia and acidosis. It is both regulated by and functionally implicated in adaptive pathways induced by these physiological stresses in tumor microenvironment. Particularly with increasing knowledge on contribution of acidosis to all key steps of cancer progression, it is becoming apparent that even those attributes of CA IX that were previously thought to be unrelated to its catalytic activity, such as disruption of E-cadherin-related cell-cell contacts, formation of focal adhesion contacts, endocytosis, and possibly others, are linked to low pericellular pH. It is now evident that CA IX provides selective advantage to cancer cells by conferring them the ability to survive hostile conditions, acquire metastatic propensity and gain resistance to both conventional and innovative therapies. Cancer cells expressing CA IX generally represent the most aggressive fraction of tumor tissue (with exception of ccRCC), and thus, CA IX–based stratification with tissue and serum biomarkers or imaging, followed by targeting of CA IX-expressing tumors, are anticancer strategies that are worth following, as supported by a number of preclinical models and clinical experiences.

Obviously, elimination of CA IX–positive cells alone might not be sufficient to achieve full and sustainable therapeutic effects against diverse tumor types and disease progression stages. However, recent era of combination therapies offers numerous opportunities for targeting CA IX in tumors that do not respond to existing therapeutic regimens. We definitely need to explore various scenarios of these combined approaches for benefit of cancer patients.
